# CA 19-9 Pancreatic Tumor Marker Fluorescence Immunosensing Detection via Immobilized Carbon Quantum Dots Conjugated Gold Nanocomposite

**DOI:** 10.3390/ijms19041162

**Published:** 2018-04-11

**Authors:** Nawal Ahmad Alarfaj, Maha Farouk El-Tohamy, Hesham Farouk Oraby

**Affiliations:** 1 Department of Chemistry, College of Science, King Saud University, P.O. Box 22452, Riyadh 11495, Saudi Arabia; nalarfaj@ksu.edu.sa; 2 General Administration and Medical Affairs, Zagazig University, Zagazig 44511, Egypt; 3 Department of Agronomy, Faculty of Agriculture, Zagazig University, Zagazig 44511, Egypt; heshamoraby@gmail.com

**Keywords:** carbon quantum dots, tumor markers, gold nanocomposite, CA 19-9 antigen, immunoassay technique

## Abstract

The clinical detection of carbohydrate antigen 19-9 (CA 19-9), a tumor marker in biological samples, improves and facilitates the rapid screening and diagnosis of pancreatic cancer. A simple, low cost, fast, and green synthesis method to prepare a viable carbon quantum dots/gold (CQDs/Au) nanocomposite fluorescence immunosensing solution for the detection of CA 19-9 was reported. The present method is conducted by preparing glucose-derived CQDs using a microwave-assisted method. CQDs were employed as reducing and stabilizing agents for the preparation of a CQDs/Au nanocomposite. The immobilized anti-CA 19-9-labeled horseradish peroxidase enzyme (Ab–HRP) was anchored to the surface of a CQDs/Au nanocomposite by a peptide interaction between the carboxylic and amine active groups. The CA 19-9 antigen was trapped by another monoclonal antibody that was coated on the surface of microtiter wells. The formed sandwich capping antibody–antigen–antibody enzyme complex had tunable fluorescence properties that were detected under excitation and emission wavelengths of 420 and 530 nm. The increase in fluorescence intensities of the immunoassay sensing solution was proportional to the CA 19-9 antigen concentration in the linear range of 0.01–350 U mL^−1^ and had a lower detection limit of 0.007 U mL^−1^. The proposed CQDs/Au nanocomposite immunoassay method provides a promising tool for detecting CA 19-9 in human serum.

## 1. Introduction

Cancer is a striking public health problem and a major cause of death worldwide [1]. Since time is a critical aspect in the life of cancer patients, diagnosing tumors at an early stage generally increases survival chances [2]. It has been documented that elevated levels of several biological and biochemical molecules—termed biomarkers (i.e., enzymes, antibodies, nucleic acids, carbohydrates, peptides, hormones, and metabolites and biological processes such as apoptosis and proliferation)—are correlated with various disease conditions [3]. In cancer clinical practice, biomarkers are used for screening and diagnosis, as well as predicting prognosis, progression, disease recurrence, and monitoring of therapy outcome [4,5]. Elevated levels of carbohydrate antigen 19-9 (CA 19-9) in blood plasma is one of the warning indicators of pancreatic cancer, an aggressive type of cancer that is seldom diagnosed at an early stage. These high levels of CA 19-9 are also associated with other cancer and non-cancer cases, such as digestive tract, breast, lung, and ovarian cancers, as well as pancreatitis and obstructive jaundice [6]. Even though CA 19-9 is the only U.S. Food and Drug Administration (FDA) approved biomarker for pancreatic cancer, its low accuracy, sensitivity, and specificity with increased false positives present a great challenge [7]. Thus, a sensitive, specific, and practical technique for the clinical detection of trace levels of CA 19-9 in human serum would be an advantageous strategy for enhancing the efficiency of developing pancreatic cancer treatment protocols [6].

Several techniques have been developed to detect CA 19-9, such as enzyme-linked immunosorbent assay [8], chemiluminescent enzyme [9] and electrochemical immunoassays [10], and chemiluminescent immunosensors [11] and quantum dot-labeled immunosensors [12,13,14]. Although most of these methods have provided acceptable detection limits of CA19-9, drawbacks related to time, expense, and specificity are still a concern.

Nanotechnology is explosively growing and giving infinite benefits in almost all fields of our life. Among these are medicinal applications [15], catalysis [16], drug delivery [17], drug analysis [18], tissue engineering [19], cell imaging [20] and cancer biomarkers [21]. Moreover, eco-friendly green-synthesized nanoparticles that avoid the usage of toxic reagents, reducing the usage of chemical reagents and analytical instruments, are dramatically increasing as well [22,23]. 

Carbon quantum dots (CQDs) (luminescent semiconductor carbon nanoparticles) present unique optical, physicochemical, and electronic properties. According to the employed synthesis methodology, it is possible to prepare CQDs with desirable sizes and specific emission properties. They demonstrate great potential to transduce luminescent signals with high stability, high quantum yield, high emission tenability, biocompatibility, and chemical inertness [24]. Thus, the discovery of CQDs has triggered extensive studies to exploit these fluorescence properties in several analytical applications such as optoelectronic [25], photocatalysis [26], photoluminescent [27], bioimaging and biosensing [28], and immunoassay detection [29,30]. Additionally, owing to their abilities as electron donors and/or acceptors, CQDs have been added as reducing and stabilizing agents in the synthesis of metallic gold (Au) and silver (Ag) nanocomposites [31] for sensing applications [32,33]. Generally, the earlier reports on Au/CQDs nanocomposites were published in 2013 and 2014. The synthesis of these nanocomposites was performed using one step or several steps but in most cases, the gold nanoparticles (AuNPs) and CQDs were modified separately [34,35,36,37]. AuNPs/CQDs nanocomposites have many benefits. They are used in the fields of immunoassay and gene detection [38,39], electrocatalytic analysis [40], and biosensors [41,42]. 

Horseradish peroxidase (HRP) has been a powerful tool that is commonly used for labeling antibodies in immunosensing or sandwich capping measurements. It has been used in competition reactions with the unlabeled analyte in the sample to bind with antibodies. It has the ability to convert colorless molecules into colored or fluorescent moieties, providing higher sensitivity for the detection of an analyte. It is used in electrochemical biosensors [43] and industrial analysis [44]. Due to its availability in a relatively pure form and its high stability, it is widely used in immobilized immunoassay probes for antibody–antigen reactions [45]. 

In the present work, a new, simple, and fast immunoassay fluorescence sensing system based on CQDs/Au nanocomposite was developed for the detection of CA 19-9, a pancreatic cancer biomarker, in human serum. Glucose-derived CQDs were employed as the reducing material, as well as stabilizing agent, for the easy synthesis of CQDs/Au nanocomposite. The as-prepared CQDs/Au nanocomposite was immobilized by CA 19-9 monoclonal antibodies labeled with the horseradish peroxidase (Ab–HRP) enzyme. 

## 2. Results and Discussion

### 2.1. Characterization of CQDs

**Figure 1:**
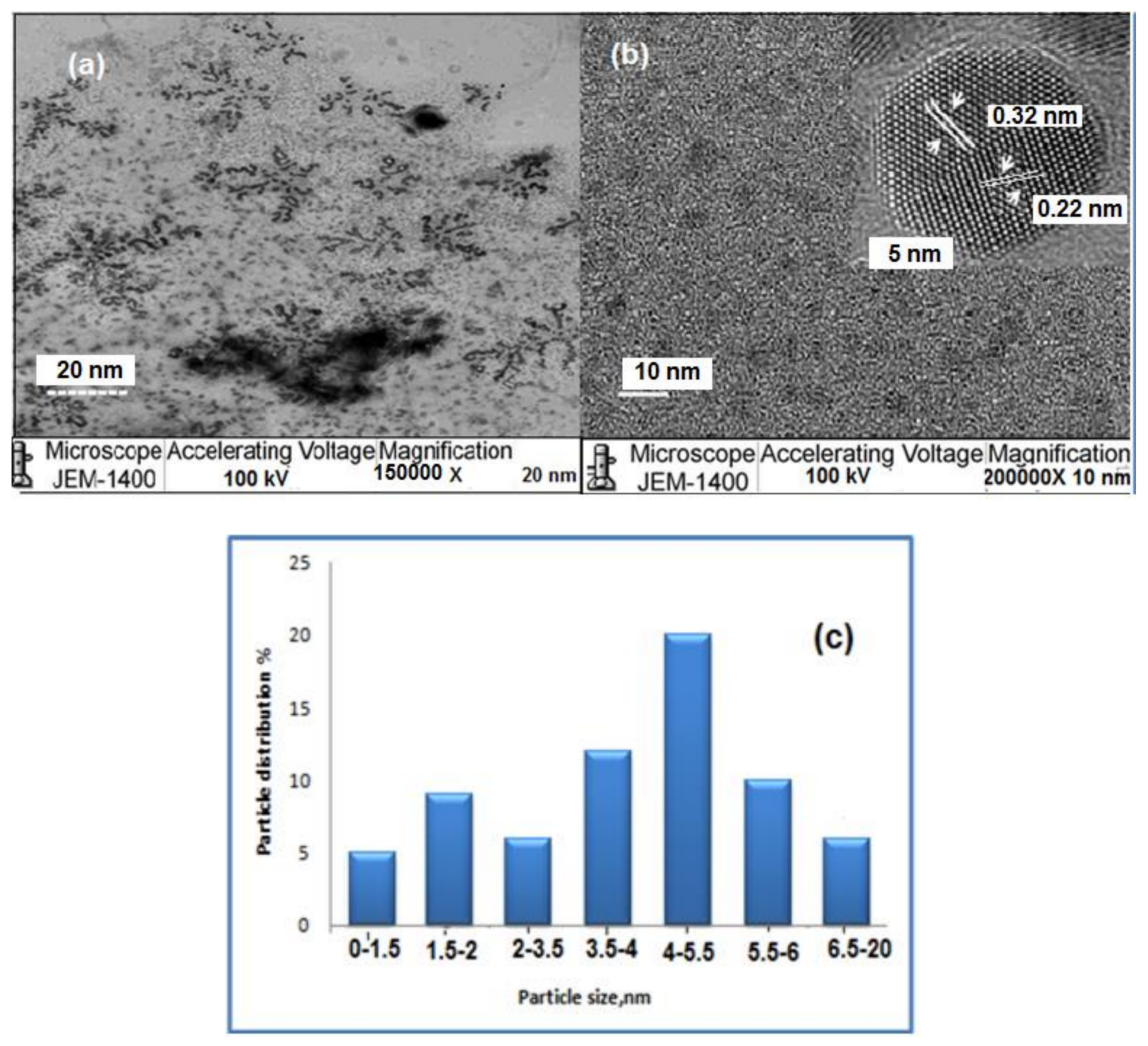
(**a**) TEM image of carbon quantum dots (CQDs), (**b**) High-resolution transmission electron microscopy (HRTEM) image of the spherical morphology of mono-dispersed particles with an average diameter of 5 nm and (**c**) the particle size distribution curve of the CQDs.

The morphological evaluation of the obtained CQDs was accomplished by TEM. To prepare the samples for detection under TEM, approximately 4 µL of the CQDs suspension was dropped on the TEM carbon grid. The recorded image (Figure 1a) revealed mono-dispersed, spherical CQDs with uniform distribution. The high-resolution transmission electron microscopy (HRTEM) showed CQDs with a uniform size less than 10 nm (Figure 1b). The calculated particle size distribution curve showed an average particle size of 1.5 ± 0.2 to 5.0 ± 0.2 nm (Figure 1c). A dynamic light scattering (DLS) test also confirmed that the average particle size varied between 10 ± 0.6 and 20 ± 0.6 nm. The difference in size between these two measurements arises from the phenomena and mechanism involved in the respective experiments. Moreover, due to DLS studies usually taking into account the hydration dynamics of the resultant particles, it shows a higher value than TEM. The studies clarified the formation of fluorescent CQDs that emit a green color on exposure to UV light, which could be further immobilized by HRP using a peptide bond formation [43].

**Figure 2:**
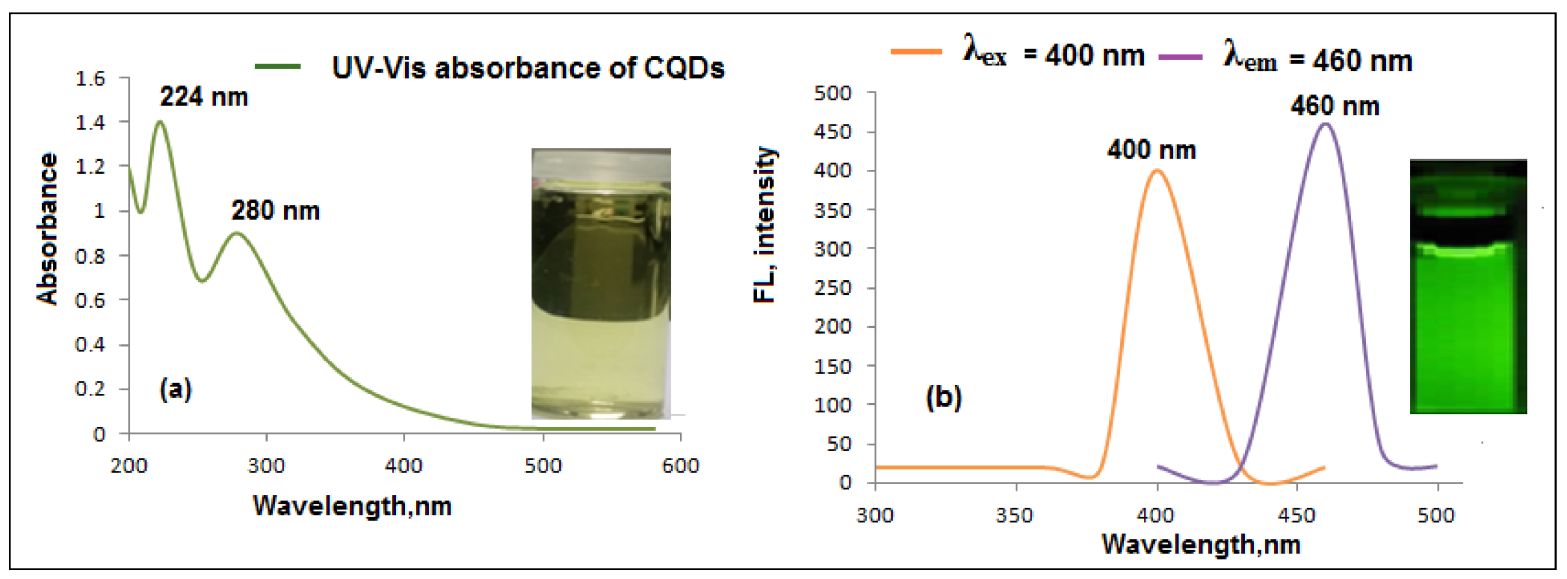
Spectroscopic detection of CQDs. (**a**) UV-Vis spectrophotometric absorption spectrum of CQDs and (**b**) spectrofluorimetric detection of CQDs at λ_ex_ = 400 and λ_em_ = 460 nm.

The UV-Vis spectrum of the CQDs recorded two significant absorption peaks at 224 and 280 nm (Figure 2a). These distinct peaks were attributed to the presence of *π* − π* transition of C=C and *n* − π* transition of the carbonyl group C=O, respectively [46]. The fluorescence spectra of CQDs were investigated and the maximum excitation and emission were recorded at λ_ex_ = 400 and λ_em_ = 460 nm, respectively (Figure 2b).

### 2.2. Characterization of CQDs/Au Nanocomposite

**Figure 3:**
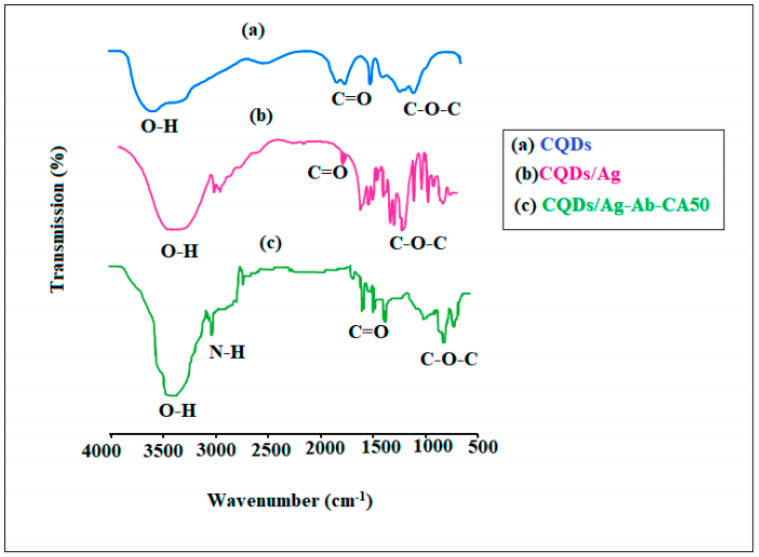
FT-IR spectra of (**a**) CQDs, (**b**) carbon quantum dots/gold (CQDs/Au) nanocomposite and (**c**) immobilized CQDs/Au–Ab–HRP.

Microscopic and spectroscopic techniques were carried out to confirm the characterization of the CQDs/Au nanocomposite after and before immobilization with Ab–HRP. As depicted in Figure 3a,b, the FT-IR spectrum displayed the appearance of different peaks that represent some functional groups located on the surface of CQDs, such as the stretching vibration of C–OH (3300 cm^−1^) and C–H (2920 cm^−1^). Additionally, vibrational absorption bands corresponding to C=O, amide III, and C–O–C were recorded at 1750 cm^−1^, 1381 cm^−1^, and 1250 cm^−1^, respectively. It is worth mentioning that the presence of –OH groups with their reducing activity on the surface of CQDs confirmed the ability of CQDs to donate electrons during the formation reaction of the CQDs/Au nanocomposite. Moreover, the presence of COOH groups on the surface of CQDs, which possess a strong affinity towards Au ions, facilitates the adsorption of Au ions on the CQD surface and prevents their aggregation. Therefore, CQDs can be used as reducing and stabilizing agents in the formation of the CQDs/Au nanocomposite. New peaks were also observed at 3143 cm^−1^ and at 1678 cm^−1^ of stretching vibration of N–H and C=O, confirming the formation of peptide bonds and denoting the immobilization of the Ab–HRP on the surface of the CQDs/Au nanocomposite (Figure 3c).

**Figure 4:**
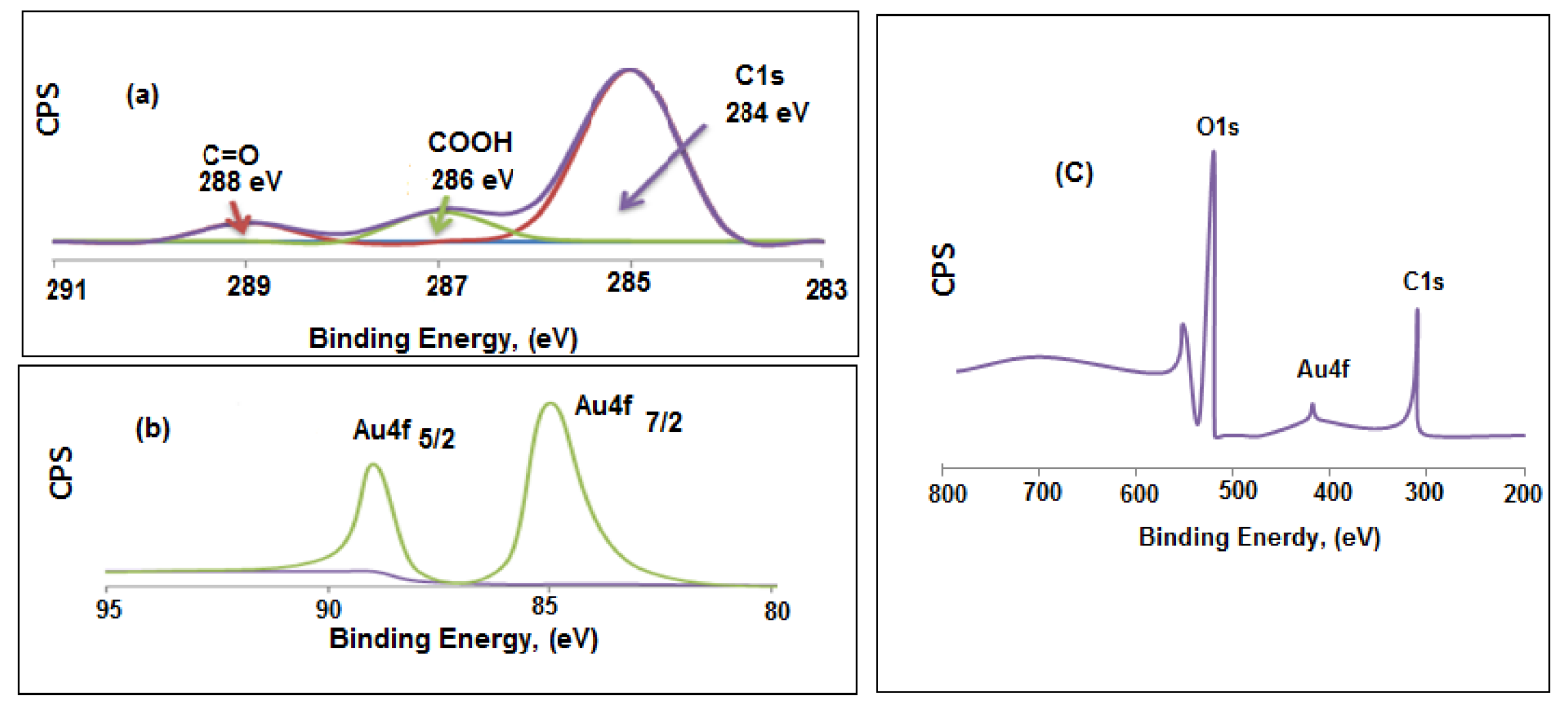
X-ray photoelectron spectroscopy (XPS) spectra of (**a**) CQDs (**b**) Au (**c**) CQD/Au nanocomposite.

The X-ray photoelectron spectroscopy (XPS) spectra of the CQDs were recorded (Figure 4a) and demonstrated that the as-synthesized CQDs have certain functional groups such as C=O and C–O–C with binding energies of 288 and 286 eV, respectively. Two more distinct peaks at binding energies 87 and 85 eV were recorded, which were attributed to Au4f_5/2_ and Au4f_7/2_, respectively (Figure 4b). Additionally, the high-resolution XPS showed the presence of C1s, Au4f, and O1s peaks at binding energies of 378, 484, and 530 eV, respectively (Figure 4c). The obtained XPS spectrum confirmed that the CQD surfaces were coated with Au nanoparticles, forming a CQDs/Au nanocomposite.

**Figure 5:**
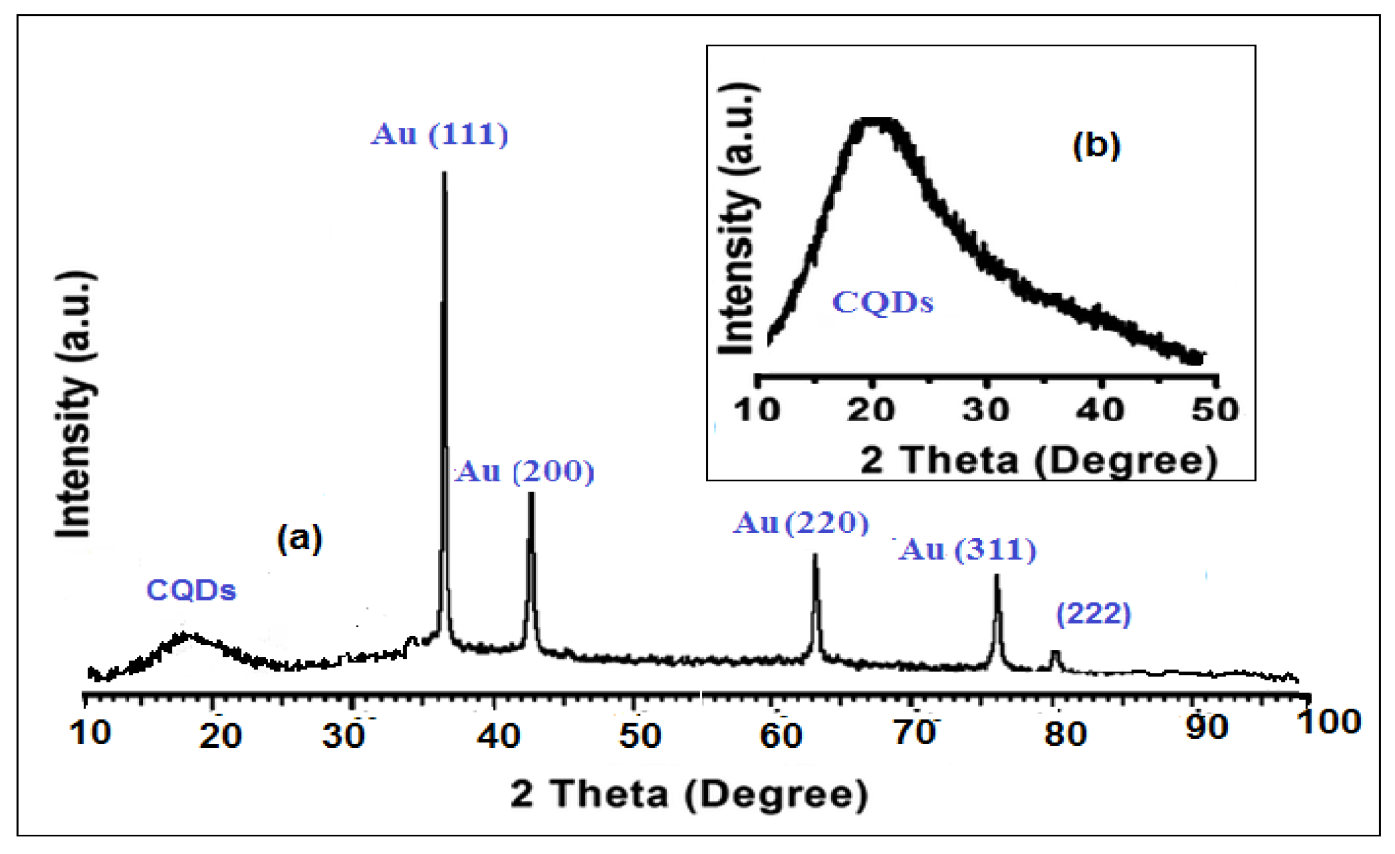
XRD pattern of (**a**) CQDs and (**b**) CQDs/Au nanocomposite.

XRD patterns of CQDs and CQDs/Au nanocomposite were recorded. It was observed that the CQDs in the CQDs/Au nanocomposite was hardly distinguished (Figure 5a) when compared with the XRD pattern of CQDs (Figure 5b) that exhibit a single broad (002) peak at 19° (2θ). This reflects an increase in the interlayer spacing of the CQDs as a result of the growth of Au nanoparticles on the surface of the CQDs. In addition, the XRD pattern of the CQDs/Au nanocomposite revealed the presence of significantly different peaks at 38.1°, 43.5°, 64.0°, and 77.1° (2θ), indicating the distribution of Au (111), Au (200), Au (220), and Au (311), respectively, on the surface of the CQDs. 

**Figure 6:**
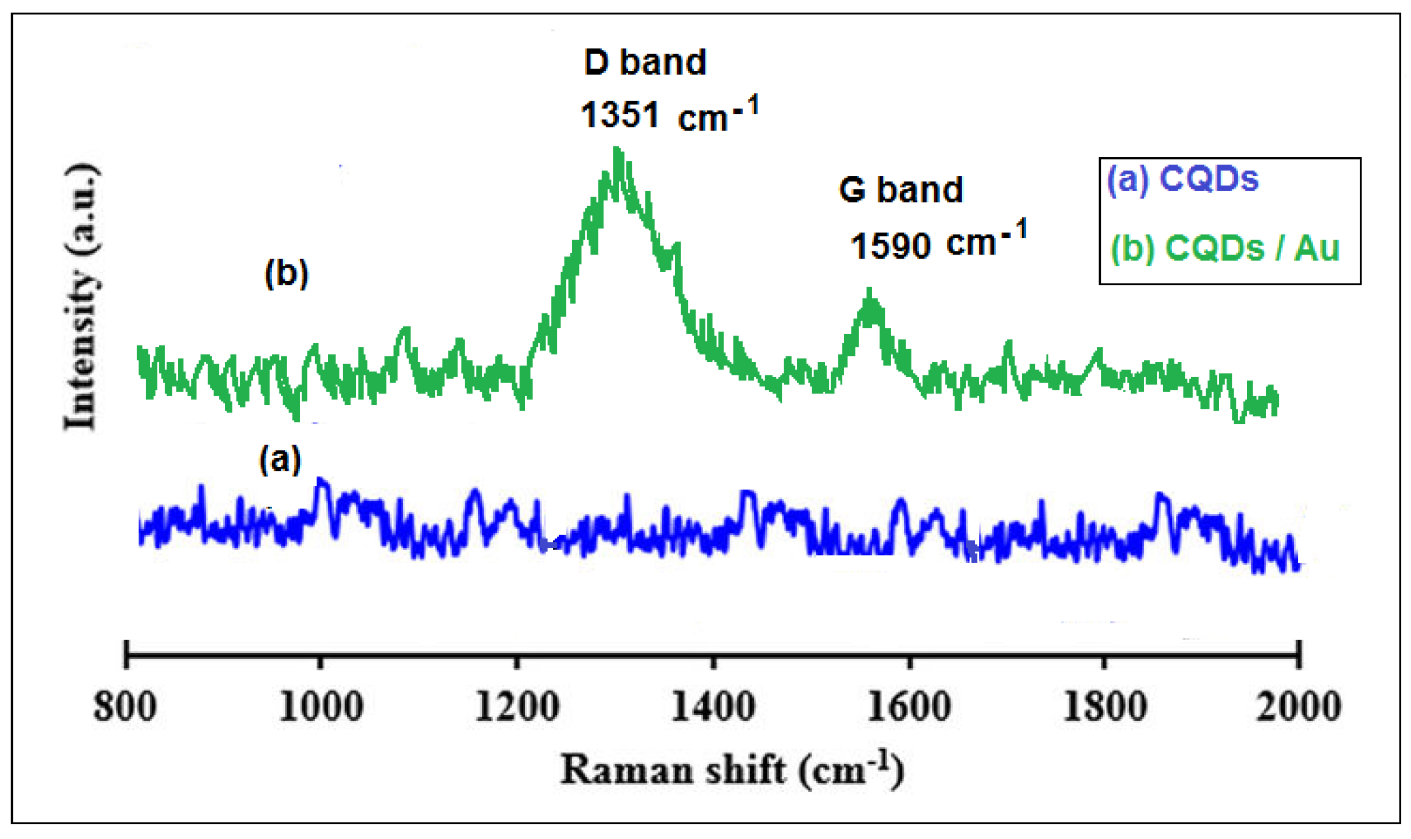
Raman spectra shift of (**a**) CQDs and (**b**) the CQDs/Au nanocomposite.

Raman spectrum of the as-prepared CQDs/Au nanocomposite was recorded. Due to the strong fluorescence background of the CQDs, they did not exhibit any Raman signal (Figure 6a). However, the recorded signals of the CQDs/Au nanocomposite showed two distinct peaks at 1351 cm^−1^ and 1590 cm^−1^, which correspond to the typical D and G bands of carbon nanoparticles (Figure 6b). The successful formation of the CQDs/Au nanocomposite was proven by the partial quenching of CQDs fluorescence background and the appearance of the two previously mentioned signals. 

### 2.3. Optimization of Fluorescence Immunoassay Detection Conditions

In order to select suitable conditions for maximum sensitivity of the proposed immunoassay technique, various parameters were studied and optimized. These parameters included several experimental variables such as the amount of immobilized CQDs/Au–Ab–HRP nanocomposite, the buffer concentration, pH, and the pre-incubation time for the immunoassay reaction between the targeted CA 19-9 in serum samples and both Ab–CA 19-9 and immobilized CQDs/Au–Ab–HRP nanocomposite. 

**Figure 7:**
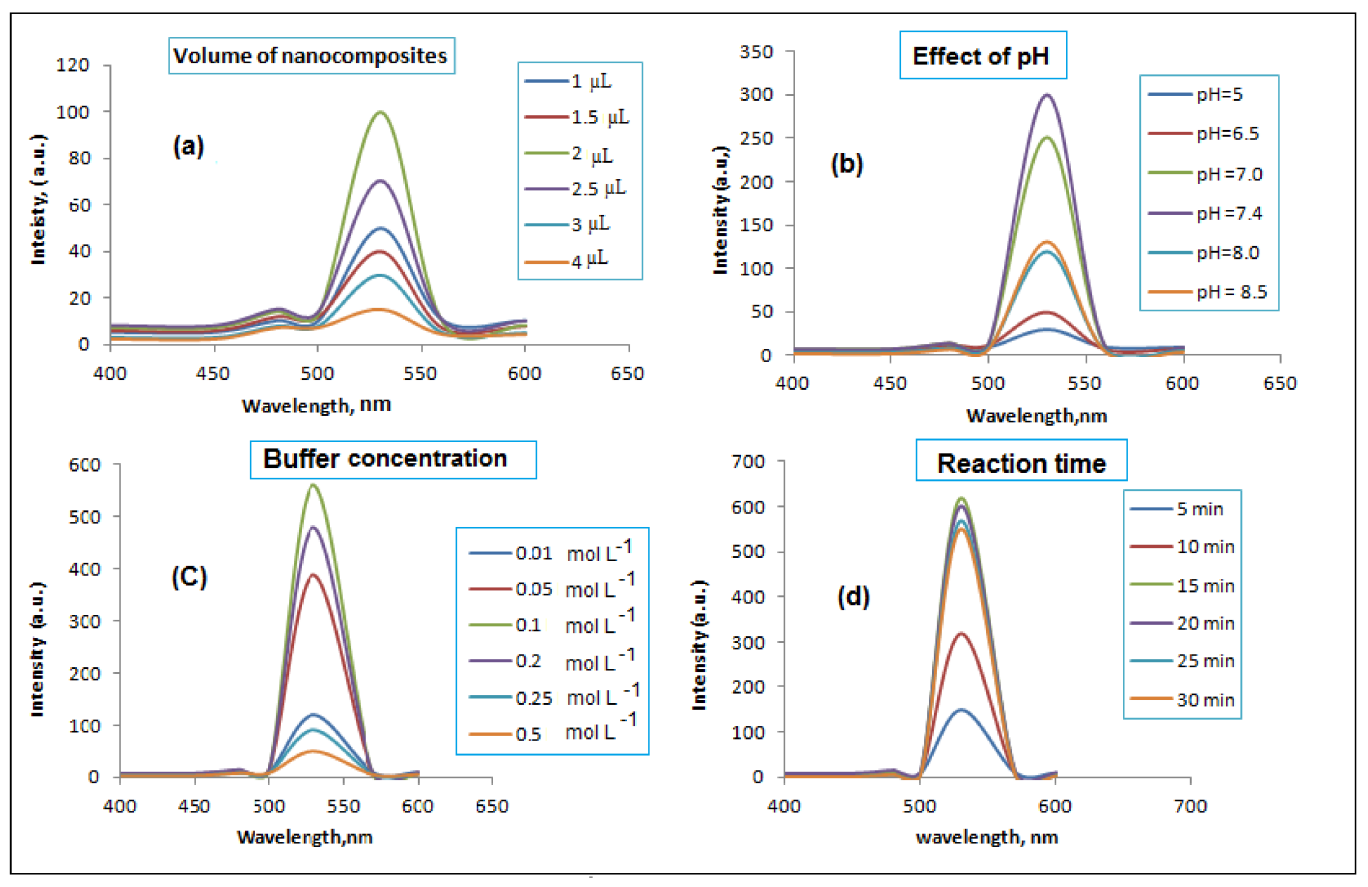
Optimization of fluorescence detection conditions at λ_ex_ = 420 and λ_em_ = 530 nm: (**a**) effect of nanocomposite volume, (**b**) effect of pH value, (**c**) effect of buffer concentration, (**d**) effect of reaction time.

The suitable amount of the immobilized CQDs/Au–Ab–HRP nanocomposite was selected by testing different amounts in the range of 1.0–4.0 µL. It was observed that the maximum fluorescence intensity was obtained by adding 2.0 µL of the immobilized CQDs/Au–Ab–HRP nanocomposite (Figure 7a).

The fluorescence intensity as a function of different phosphate buffer pH environments was studied. The emission intensity was recorded by varying the pH of the phosphate buffer from 5–8.5. The peak intensity changed slightly with changing the pH values. The maximum peak intensity was recorded at a pH value of 7.4. At pH less than 7.4, the fluorescence intensity was decreased, which can be attributed to the instability of the CQDs/Au nanocomposite in an acidic medium or the aggregation of Au nanoparticles (Figure 7b).

The effect of buffer concentration on the peak intensity was investigated using the phosphate buffer in a concentration range of 0.01–0.5 mol L^−1^ (Figure 7c). The fluorescence peak intensity was elevated by increasing the buffer concentration to 0.1 mol L^−1^. At high buffer concentrations, the peak intensity gradually decreased due to the aggregation of the CQDs/Au nanocomposite.

The immunoreaction time was examined by repeating the analytical process using a reaction time in the range of 5–30 min. The suitable incubation time to complete the immunoreaction between the investigated CA 19-9 antigen and the immobilized CQDs/Au–Ab–HRP was 15 min (Figure 7d). 

### 2.4. Analytical Figures of Merit

**Figure 8:**
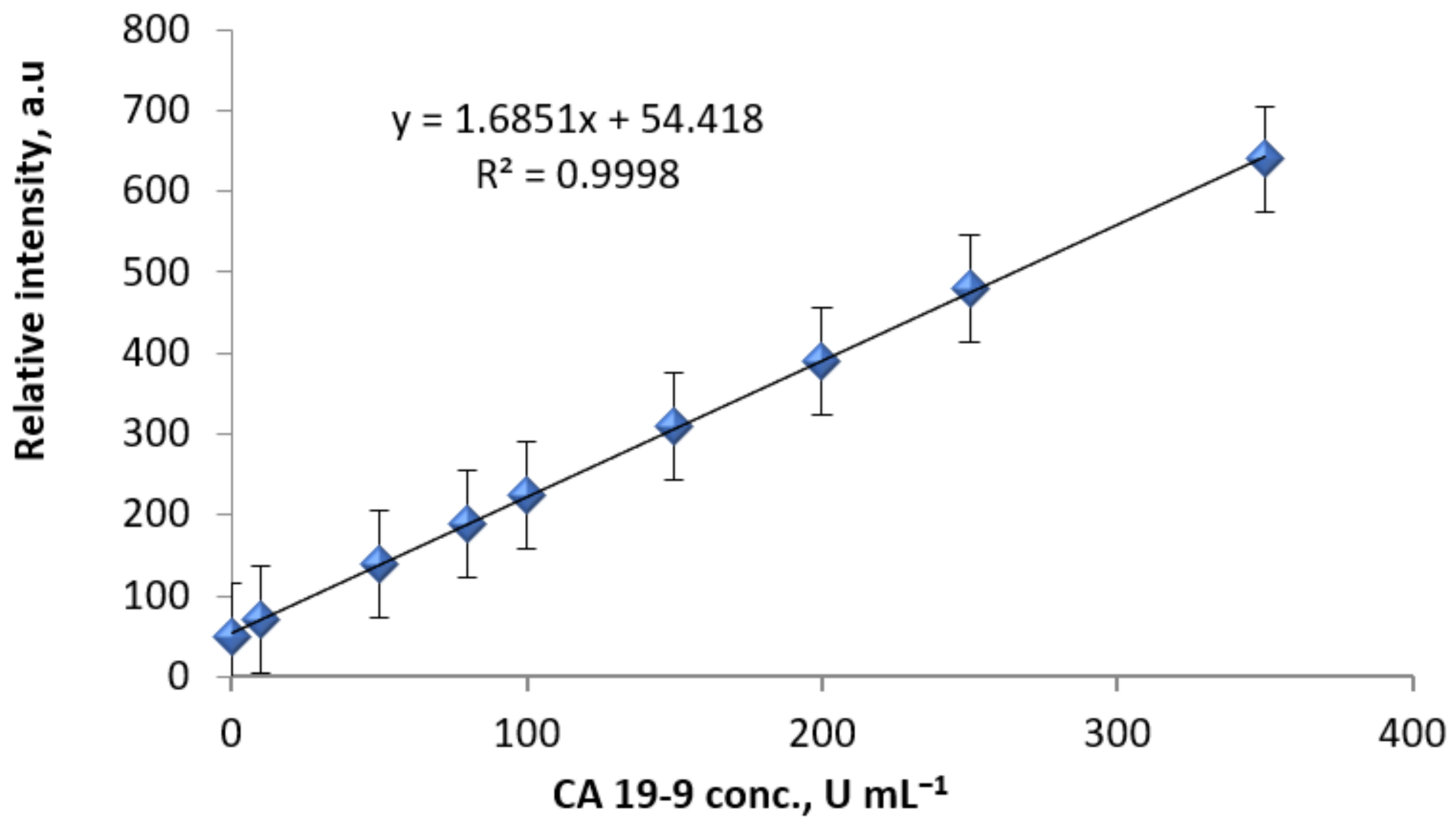
A calibration graph for the detection of CA 19-9 antigen using immobilized CQDs/Au–Ab–HRP immunoassay fluorescence method.

After optimizing the analytical conditions, the suggested immobilized CQDs/Au–Ab–HRP immunoassay method was employed for the detection of CA 19-9 antigen in nine serum samples and the analytical figures of merit were obtained. The plotted calibration graph was linear over a concentration range of 0.01–350 U mL^−1^ with a limit of detection of 0.007 U mL^−1^ (Figure 8). The applied regression equation was *I_F_* = 1.6851C + 54.418, (*r*^2^ = 0.9998) and the relative standard deviation percentage (RSD%) of six replicate measurements equaled 1.4%. The obtained results revealed that the suggested method possesses a high sensitivity, good stability, and acceptable linearity.

### 2.5. Suitability of CQDs/Au–Ab–HRP Immunoassay Technique

To evaluate the suitability of the suggested immunoassay method for the detection of CA 19-9 antigen in human serum, a comparative study between the suggested method and previously published approaches were carried out. The suggested approach demonstrated a strong advantage when compared with the previously reported techniques, particularly with respect to its simplicity, its easiest to carry out, and that no hazardous chemicals are required. Furthermore, the recorded results indicated a high sensitivity and displayed rapid detection of the investigated CA 19-9 antigen with a lower detection limit of 0.007 U mL^−1^ compared with the other recommended approaches (Table 1). 

### 2.6. Accuracy and Precision of CQDs/Au–Ab–HRP Immunoassay Technique

The accuracy of the proposed immunoassay method was evaluated by carrying out an analysis of 15 serum samples using the proposed technique. The obtained results were compared with another previously published method [11]. This method was mainly based on the chemiluminescent detection of CA 19-9 antigen using a cross-linked chitosan membrane. The comparative data reported between the two methods revealed acceptable agreement (Table 2). 

The precision of the suggested immunoassay method was determined by employing the inter-day and intra-day assay. Three different determinations of one sample were recorded for intra-day precision through three successive occasions. For the inter-day assay, three determinations for one sample were recorded during three successive days. The used CA 19-9 concentration was 10 U mL^−1^ and the calculated percentage (%) RSDs were 1.6 and 1.2% for inter-day and intra-day assays, respectively. The obtained results revealed a strong, precise technique. 

### 2.7. Study of Possible Interferences

To study the selectivity of the developed immunoassay method, the influence of some possible cations such as Na^+^, K^+^, Ca^2+^, Mg^2+^, Zn^2+^, Ag^+^, and Ba^2+^ were investigated in the presence of 10 U mL^−1^ CA 19-9. Additionally, some possible sugars, including glucose, sucrose, and lactose were tested. Also, different amino acids such as glycine, cysteine, alanine, histidine, and valine were examined. Moreover, the immunoassay fluorescence method was investigated towards some compounds such as ascorbic acid, uric acid, and caffeine. Additionally, the interference of some related tumor markers including CA 27-29, CA 15-3, CA 125, and prostate specific antigen (PSA) were investigated. The summarized results in Table 3 revealed that most of the investigated species did not interfere with the detection of the targeted CA 19-9 antigen. Thus, the suggested method is highly selective towards the detection of CA 19-9 in serum samples.

### 2.8. Analysis of Real Samples

The proposed fluorescence immobilized CQDs/Au–Ab–HRP immunoassay technique was employed to detect the percentage (%) recoveries of different CA 19-9 antigen concentrations in real human serum. The blood samples were randomly collected from healthy volunteers, allowed to clot, and then centrifuged. The supernatants were subsequently detected by the proposed method with respect to the relation between the fluorescence intensity as a function of CA 19-9 concentration. Certain increments of CA 19-9 antigen were added to the above samples and the peak intensities were recorded. The percentage (%) relative standard deviations were calculated after six determinations as summarized in Table 4. The percentage (%) recoveries ranged from 97.2–99.9% and the percentage (%) relative standard deviations were 0.5–2.1%. The obtained data were assessed statistically and then compared with those obtained from another previously published method [11] using the Student’s *t*-test and *F*-test. 

## 3. Experimental

### 3.1. Instrumentation

CQDs and CQDs/Au nanocomposite UV-Vis spectra were measured using Ultrospec 2100-Biochrom spectrophotometer (Biochrom Ltd., Cambium, Cambridge, UK). The detection was carried out using 1.0 cm standard quartz cells. Fluorescence spectra of the luminescent sensing solutions were measured using Biotek Synergy H1 multi-mode reader (Biotek, Tokyo, Japan). Transmission electron microscopy (TEM) with the JEOL model 1200EX instrument (JEOL Ltd., Freising, Germany) was used to evaluate the size and shape of the synthesized CQDs/Au nanocomposite. X-ray powder diffraction (XRD) pattern was obtained using Siemens D-5000 diffractometer (Siemens, Erfurt, Germany) and Fourier transform infrared (FT-IR) spectra were measured using Perkin Elmer FT-IR spectrophotometer (PerkinElmer Ltd., Yokohama, Japan). X-ray photoelectron spectroscopy (XPS) and Raman spectra were recorded using Kratos Axis Ultra X-ray spectroscopy system (Kratos Analytical Ltd., Manchester, UK) and micro-Raman spectrometer (CRAIC Technologies, CA, USA), respectively.

### 3.2. Chemicals and Reagents

In the present work, all reagents were of analytical grade. Deionized water was obtained from SG-2000-10090 (Barsbuttel, Germany) and used throughout all experiments. Glucose, ammonia (NH_3_), *N*-hydroxysuccinimide (NHS), 1-ethyl-3-(3-dimethylaminopropyl) carbodiimide hydrochloride (EDC), luminol, hydrogen peroxide, chitosan of low molecular weight, and HAuCl_4_·3H_2_O (98%) were purchased from Sigma-Aldrich (Hamburg, Germany). CA 19-9 antigen, monoclonal CA 19-9 antibodies, and CA 19-9 monoclonal antibodies labeled with horseradish peroxidase (Ab–HRP) enzyme were acquired from Abcam (Cambridge, UK). Monosodium phosphate, disodium phosphate, and sodium hydroxide were obtained from BHD Ltd. Co. (Poole, UK). Multi-Serum Normal, Randox Laboratories, Northern Ireland-UK supplied the serum samples. In the real sample application, blood samples were collected from healthy volunteers after obtaining informed consent prior to the start of the study. The study was approved by research ethics committee, KSU-REC-002-E (31 December, 2017), King Saud University, KSA.

### 3.3. Synthesis of CQDs

**Scheme 1:**
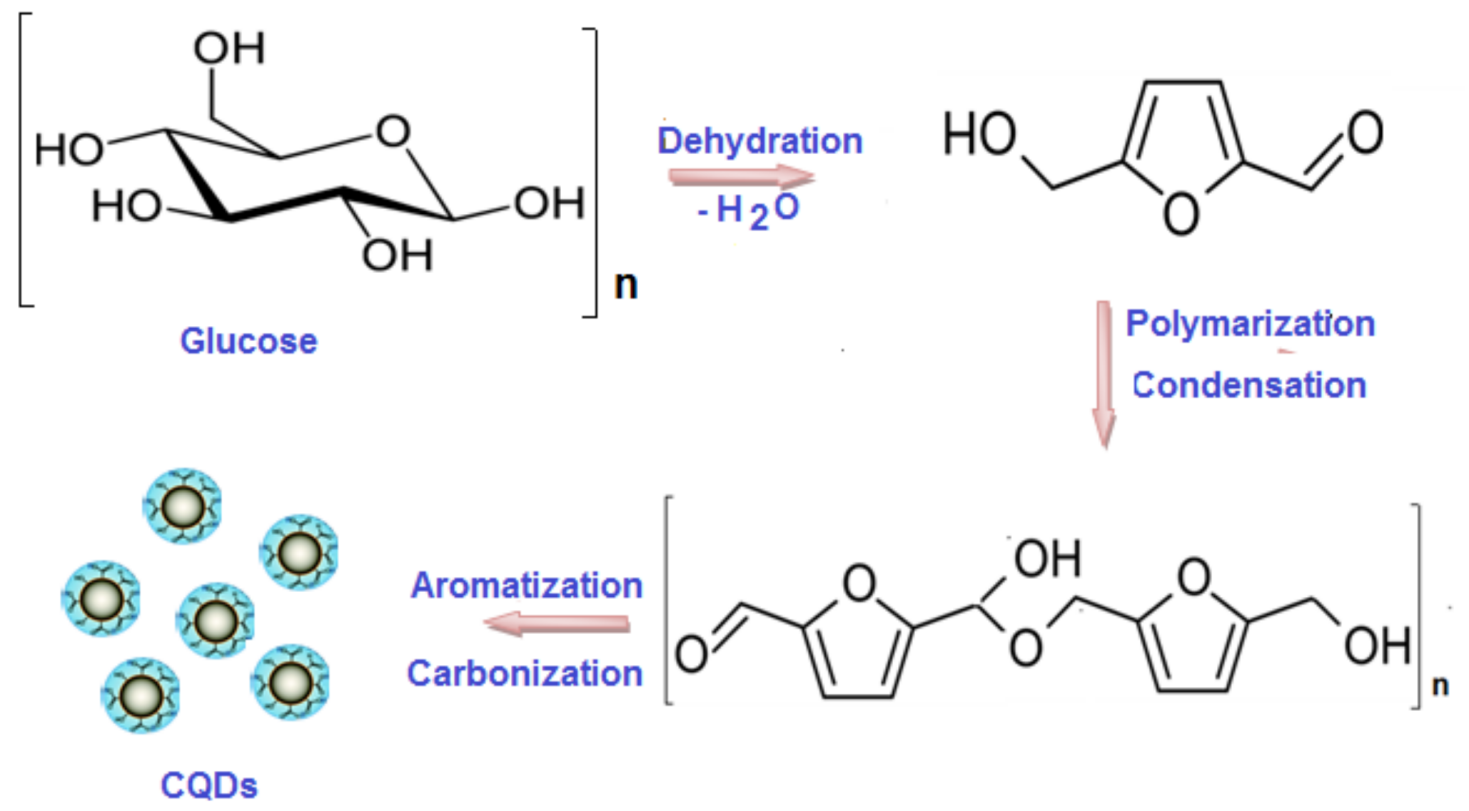
Steps in the thermal dehydration of glucose to form CQDs using a microwave-assisted method.

To prepare CQDs, an eco-friendly green synthesis method was used. It was conducted by heating 10 mL of 15% aqueous glucose at 120 °C for 15 min using a conventional microwave oven of 270 W. A semisolid yellowish solution was formed revealing the formation of CQDs (Scheme 1). The obtained volume was completed to 10 mL with deionized water and purified using a dialysis membrane (MW = 2000 Da) against deionized water for 3 h. 

### 3.4. Preparation of CQDs/Au Nanocomposite

CQDs/Au nanocomposite was prepared using a simple chemical reduction method based on the addition of 5 mL CQDs solution to 3 mL of 1.0 × 10^−2^ mol L^−1^ of HAuCl_4_ solution and 1.0 mL of 5.0 × 10^−2^ mol L^−1^ NH_3_. The mixture was transferred into a 50-mL conical flask and completed to volume using deionized water. 

The mixture was then incubated at room temperature for approximately 30 min. A pink–violet color solution of the CQDs/Au nanocomposite was obtained and stored in the refrigerator for two months at 4 °C. The stability of the prepared CQDs/Au nanocomposite was assessed using UV-Vis spectrometry. The absorbance was measured at 10-day intervals at 280 nm to check the agglomeration. No significant change in absorbance was recorded, revealing the high stability of the CQDs/Au nanocomposite. 

### 3.5. Spectroscopic and Microscopic Analysis of the CQDs/Au Nanocomposite

HRTEM was used to evaluate the surface morphology and the uniformity of the as-prepared CQDs and CQDs/Au nanocomposite. The spectral features were recorded using different spectroscopic techniques, including UV-Vis, Raman, XPS, and FT-IR spectroscopy. In addition, the crystal structure of the CQDs was studied using XRD pattern. 

### 3.6. Immobilization of CA19-9 Ab–HRP Enzyme on CQDs/Au Nanocomposite

**Scheme 2:**
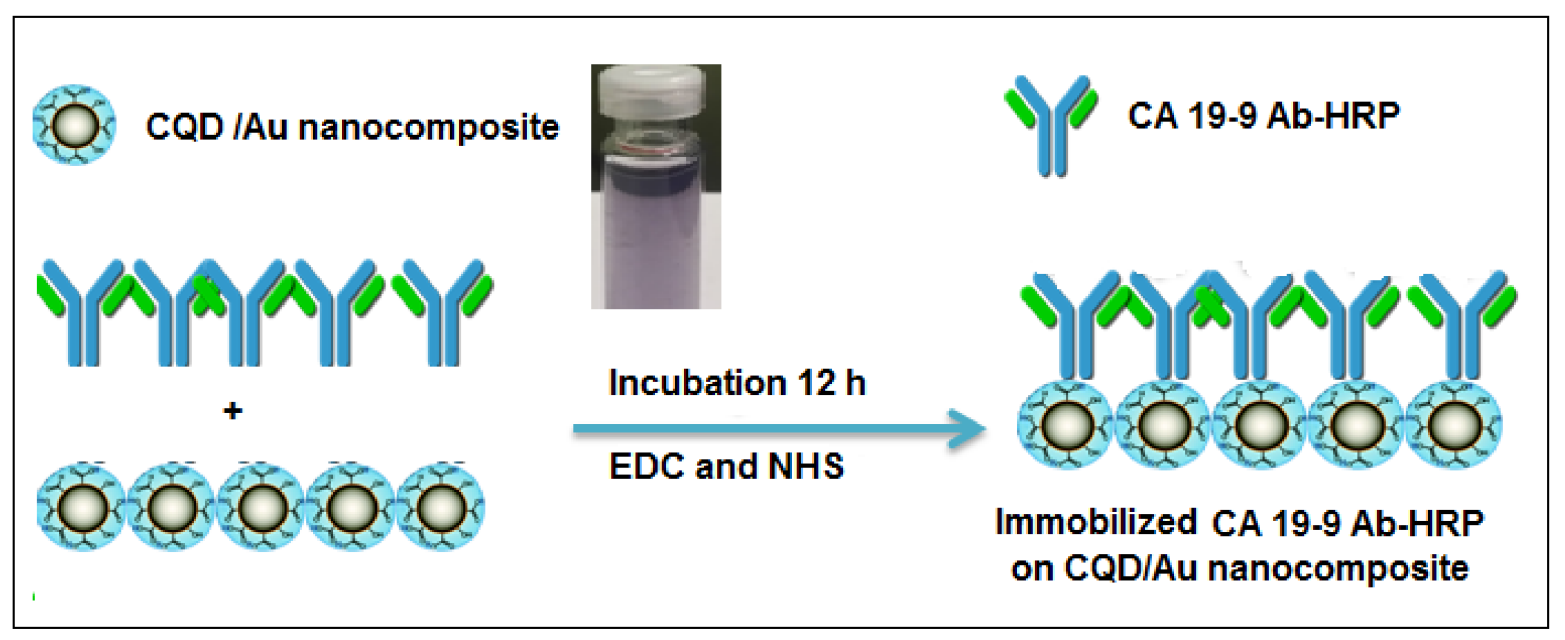
Illustration of CA 19-9 Ab–HRP immobilization on the CQDs/Au nanocomposite.

The as-prepared CQDs/Au nanocomposite was immobilized with horseradish peroxidase-labeled anti-CA 19-9 (Ab–HRP) by the formation of a peptide amide bond between the active functional carboxylic and amine groups. Under stirring, approximately 5 mL of each 2.5 × 10^−3^ mol L^−1^ EDC and 3.0 × 10^−3^ mol L^−1^ NHS was added to 5 mL of CQDs/Au nanocomposite sensing solution for 60 min. Approximately 5 mg of the CA 19-9 Ab–HRP enzyme was dissolved in 1.0 mL of 0.1 mol L^−1^ phosphate buffer (pH = 7.4) and added to the previously prepared mixture. The obtained solution was incubated for 12 h at 4 °C. CA 19-9 Ab–HRP enzyme was anchored on the CQDs/Au nanocomposite surface forming an amide linkage via a simple peptide coupling reaction (Scheme 2). Spectroscopic detection was used to confirm the immobilization of CA 19-9 Ab–HRP on the surface of the CQDs/Au nanocomposite.

### 3.7. General Principle of the Immunoassay Method

**Scheme 3:**
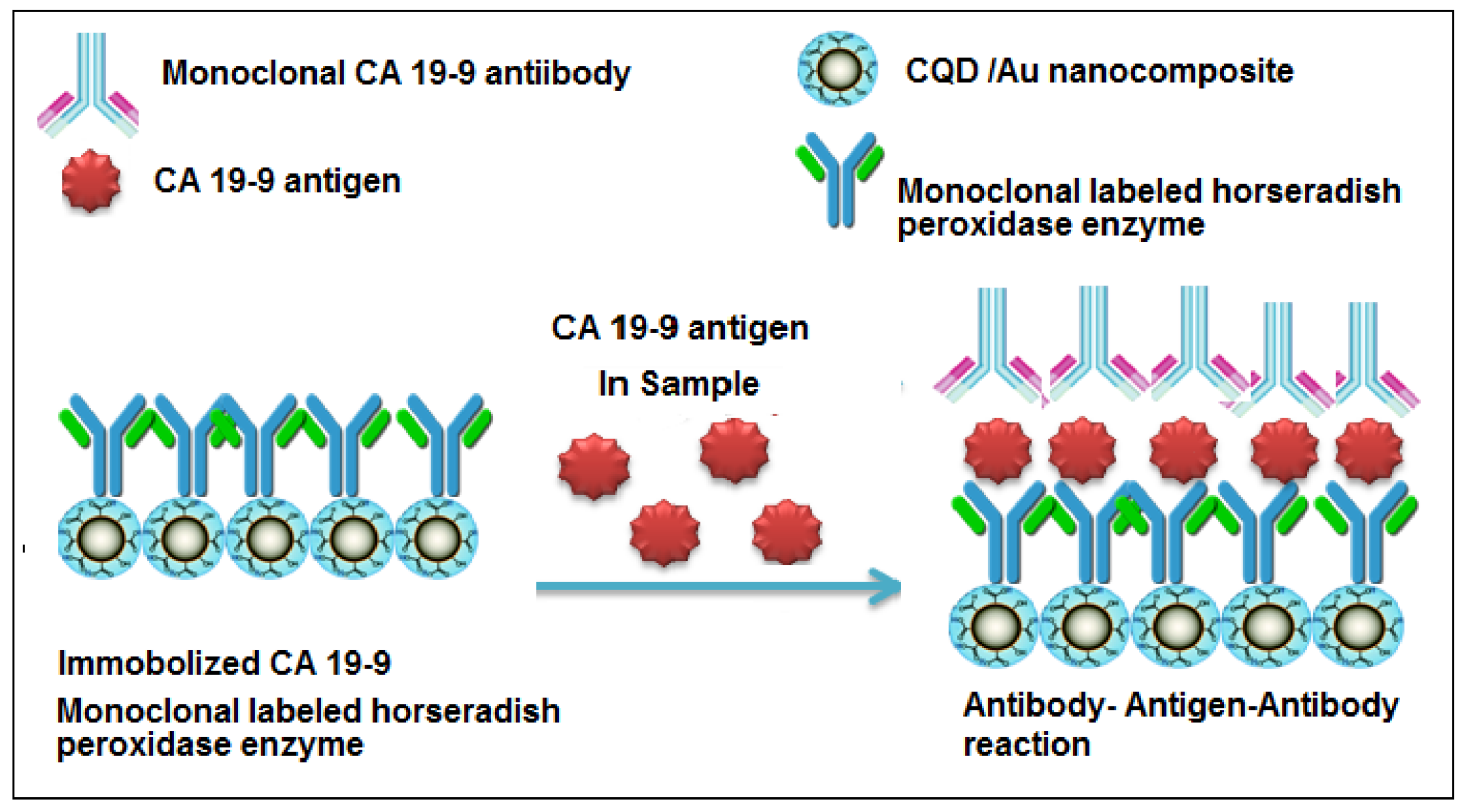
Illustration of CA 19-9 antigen sandwich capping reaction by monoclonal Ab–CA 19-9 and immobilized CQDs/Au–Ab–HRP.

This study was based mainly on the formation of stable CQDs, which have spherical particles with a size less than 10 nm and active surface functional moieties. Those CQDs have the ability to form an active sensing CQDs/Au nanocomposite with high tunable photoluminescent properties, which can be immobilized by Ab–HRP. A solid-phase immunoassay reaction type was carried out by one monoclonal CA 19-9 antibody that was coated on the microtiter wells and another monoclonal labeled-HRP antibody immobilized on the CQDs/Au nanocomposite. Under optimal conditions, a sandwich capping Ab–antigen–Ab–HRP complex was obtained (Scheme 3) and the increase in signal intensity was recorded as a function of CA 19-9 concentration. 

### 3.8. The Immunoassay Procedure 

Serum samples from healthy volunteers were collected in tubes and allowed to clot for 1 h before centrifugation. The clear samples were capped and stored at 8 °C. Approximately 50 µL of the samples previously spiked with standard CA 19-9 antigen concentrations of 0.01–350 U mL^−1^ were dispensed into the desired number of microtiter wells. They were gently mixed for 25 s and incubated at 37 °C for 1 h. 

The incubation mixture was removed by emptying the plate content into a waste container and the microtiter plate was rinsed five times using distilled water. Approximately 100 µL of the immobilized CA 19-9 Ab–HRP enzyme/CQDs/Au nanocomposite was added to each well and incubated at 37 °C for another 1 h. The incubated contents were removed by emptying the plate content into a waste container and the wells were washed using distilled water. Finally, 50 µL of each serum sample and immobilized CA 19-9 Ab–HRP enzyme/CQDs/Au nanocomposite were dispensed into each well and gently mixed for 20 s. The microplate was monitored using a microtiter reader. The fluorescence intensities were recorded.

## 4. Conclusions

A new fluorescence-based immunoassay system was conducted by employing a highly sensitive CQDs/Au nanocomposite immobilized by Ab–HRP. 

The technique used simple peptide bonds to trap the targeted CA 19-9 antigen in human serum by a sandwich capping antibody–antigen–antibody reaction. The unique characteristics of CQDs—i.e., mono-dispersed, spherical shape, homogenous distribution, and emission of a green color under UV light—provide highly sensitive immunoassay detection. The stability and reproducibility of this method were confirmed by detecting the biomarker in 15 serum samples and the obtained results were in agreement with another conventional technique. However, the proposed technique is simpler and more flexible than other immunoassay systems for the detection of different biomarkers.

## Figures and Tables

**Table 1:** A comparative study between the proposed carbon quantum dots/gold (CQDs/Au) nanocomposite immunoassay technique and the other previously published approaches.

Technique	Principle	Linear Concentration Range U mL^−1^	Detection Limit U mL^−1^	Reference
Electrochemical immunoassay	Immobilization of CA 19-9 antigen with titania sol–gel on a graphite electrode	3–20	2.68	[10]
Chemiluminescent immunosensor assay	Immobilization of CA 19-9 antigen on the cross-linked membrane	2.0–25	1.0	[11]
Chemiluminescent enzyme immunoassay	Using a derivative of 1,2-dioxetane phosphate to measure alkaline phosphatase as a labeling enzyme for the detection of CA 19-9	6.0–520	0.26	[9]
ZnO quantum dots immunoassay	Employing ZnO quantum dots as an electrochemical and luminescent immunoassay	0.1–180 1.0–180	0.04 0.25	[12]
Amperometric electrochemical immunoassay	Immobilization of CA 19-9 antibody on three-dimensional ordered macroporous magnetic sensors	0.05–15.65	0.01	[13]
Proposed CQDs/Au nanocomposite immunoassay method	Using CQDs/Au nanocomposite in the detection of CA 19-9 by a solid- phase immunoassay	0.01–350	0.007	Present study

**Table 2:** Comparative analytical results obtained from the analysis of 15 serum samples using immobilized CQDs/Au–Ab–HRP immunoassay fluorescence method and reference method [11].

Samples	Immobilized CQDs/Au/HRP Method		Immobilized Chemiluminscence Cross-Linked Chitosan Method [11]	
No.	Found U mL^−1^	% RSD (*n* = 6)	Found U mL^−1^	% RSD (*n* = 6)
1	5.9	±0.2	5.4	±0.7
2	10.5	±1.1	9.5	±1.2
3	22.1	±0.9	20.3	±1.1
4	20.3	±1.4	19.3	±0.9
5	12.8	±0.6	14.3	±0.7
6	25.0	±1.2	23.2	±1.5
7	18.2	±1.5	16.4	±0.8
8	24.1	±0.8	22.5	±0.7
9	13.6	±0.3	15.8	±0.1
10	10.8	±1.4	11.6	±1.6
11	14.0	±0.4	14.1	±0.9
12	6.3	±0.8	5.8	±1.3
13	17.4	±1.1	16.2	±1.2
14	8.3	±2.1	9.5	±0.9
15	12.5	±0.9	13.8	±0.5

**Table 3:** Tolerance limits of interferences in the detection of CA 19-9 antigen in serum using fluorescence CQDs/Au–Ab–HRP immunoassay method.

Immobilized CQDs/Au/HRP Method	
Interfering species (10 *n*g mL^−1^)	Tolerable value
Na^+^, K^+^, Ca^2+^, Mg^2+^, Zn^2+^, Ag^+^, Ba^2+^	1000
Glucose, sucrose, lactose	650
Glycine, alanine, histidine, valine	420
Uric acid, ascorbic acid, caffeine	250
CA 27-29, CA 15-3, CA 125, and PSA	510

**Table 4:** Comparative quantitative determination of CA 19-9 antigen in real serum samples using fluorescence-based CQDs/Au–Ab–HRP immunoassay method and immobilized-Chemiluminescence cross-linked chitosan method [11].

Initial CA 19-9 Conc. (U mL^−1^)	Added CA 19-9 (U mL^−1^)	Immobilized CQDs/Au/HRP Method			Immobilized-CL Cross-Linked Chitosan Method [11]			*t* = (2.228) *	*F* = (5.05) *
		Detected CA 19-9 (U mL^−1^)	% Recovery (*n* = 6)	% RSD (*n* = 6)	Detected CA 19-9 (U mL^−1^)	% Recovery (*n* = 6)	% RSD (*n* = 6)	*t*-Test	*F*-Test
0.6	0.5 5.0 10.0	1.09 5.58 10.54	99.1 99.6 99.4	1.2 1.4 1.6	1.08 5.54 10.56	98.2 98.9 99.6	1.5 1.6 1.7	1.248 0.809 0.210	1.56 1.31 1.12
1.7	0.5 5.0 10.0	2.19 6.58 11.47	99.5 98.2 98.0	1.5 0.9 1.1	2.17 6.56 11.44	98.6 97.9 97.8	2.1 1.2 1.8	0.860 0.495 0.233	1.96 1.77 2.68
5.9	0.5 5.0 10.0	6.39 10.87 15.46	99.8 99.7 97.2	0.6 0.9 1.3	6.37 10.82 15.43	99.5 99.2 97.0	0.4 1.5 1.8	1.040 0.706 0.222	2.25 2.77 1.92
8.7	0.5 5.0 10.0	9.17 13.55 18.69	99.7 98.9 99.9	0.6 1.5 0.5	9.14 13.51 18.66	99.3 98.6 99.8	1.0 0.9 0.8	0.842 0.424 0.259	2.78 2.77 2.56
10.3	0.5 5.01 0.0	10.75 15.17 19.99	99.5 99.2 98.5	1.3 0.9 1.8	10.69 15.00 19.87	98.9 98.0 97.8	1.2 0.7 2.4	0.831 2.553 0.573	1.17 1.65 1.78
3.5	0.5 5.0 10.0	3.98 8.47 13.42	99.5 99.6 99.4	1.7 1.1 1.3	3.92 8.45 13.34	98.0 99.5 98.8	1.5 0.7 1.4	1.629 0.187 0.771	1.28 2.47 1.16
12.7	0.5 5.0 10.0	13.02 17.63 22.20	98.6 99.6 97.8	0.9 1.2 1.6	12.98 17.59 22.24	98.3 99.4 97.9	1.1 0.6 2.1	0.515 0.367 0.093	1.49 4.00 1.72
14.0	0.5 5.0 10.0	13.98 18.95 23.84	96.4 99.7 99.3	2.1 0.7 1.1	13.99 18.92 23.81	96.5 99.6 99.2	1.7 0.8 1.3	0.091 0.227 0.144	1.53 1.31 1.39

* Theoretical values of “*t*” and “*F*” tests at *p* = 0.05.
