# Correction: Alarfaj et al. CA 19-9 Pancreatic Tumor Marker Fluorescence Immunosensing Detection via Immobilized Carbon Quantum Dots Conjugated Gold Nanocomposite. *Int. J. Mol. Sci.* 2018, *19*, 1162

**DOI:** 10.3390/ijms26041406

**Published:** 2025-02-07

**Authors:** Nawal Ahmad Alarfaj, Maha Farouk El-Tohamy, Hesham Farouk Oraby

**Affiliations:** 1 Department of Chemistry, College of Science, King Saud University, P.O. Box 22452, Riyadh 11495, Saudi Arabia; nalarfaj@ksu.edu.sa; 2 General Administration and Medical Affairs, Zagazig University, Zagazig 44511, Egypt; 3 Department of Agronomy, Faculty of Agriculture, Zagazig University, Zagazig 44511, Egypt; heshamoraby@gmail.com

In the original publication [1], there was a mistake in Figure 3. The mistake was a discontinued part of the curve line due to using different software and picture scaling. The corrected Figure 3 appears below. The authors state that the scientific conclusions are unaffected. This correction was approved by the Academic Editor. The original publication has also been updated.

**Figure 3:**
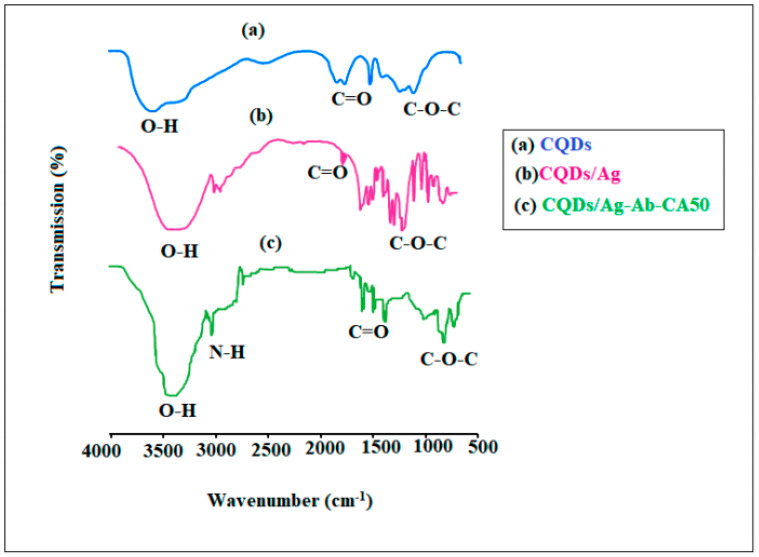
FT-IR spectra of (**a**) CQDs, (**b**) carbon quantum dots/gold (CQDs/Au) nanocomposite and (**c**) immobilized CQDs/Au–Ab–HRP.
